# Effects of a low-dose IL-2 treatment in HLA-B27 transgenic rat model of spondyloarthritis

**DOI:** 10.1186/s13075-021-02559-y

**Published:** 2021-07-16

**Authors:** L. M. Araujo, Q. Jouhault, I. Fert, I. Bouiller, G. Chiocchia, M. Breban

**Affiliations:** 1 Infection & Inflammation, UMR 1173, Inserm, UVSQ/Université Paris Saclay, 2 ave de la Source de la Bièvre, 78180 Montigny-le-Bretonneux, France; 2 Laboratoire d’Excellence Inflamex, Université Paris Descartes, Sorbonne Paris Cité, Paris, France; 3 Service de Rhumatologie, Hôpital Ambroise Paré, AP-HP, 9 ave Charles de Gaulle, 92100 Boulogne, France

**Keywords:** Low-dose IL-2, HLA-B27 transgenic rat, Regulatory T cells, Spondyloarthritis

## Abstract

**Abstract:**

**Introduction/Aim:**

HLA-B27/human β2m transgenic rats (B27-rats) develop an inflammatory disorder resembling spondyloarthritis (SpA) with dysregulated IL-10/IL-17 production by regulatory T cells (Treg). Treg plays a major role in controlling pathogenic inflammatory processes. Interleukin 2 (IL-2), a cytokine which promotes Treg cell survival and function, may thus have therapeutic efficacy in SpA. Here, we tested this hypothesis using a low dose of IL-2 treatment in B27-rat.

**Material and methods:**

B27-rats aged 4 weeks (before disease onset) and nontransgenic (NTG) littermates were administered intraperitoneally recombinant human IL-2 (Sanofi®; 2,000IU/injection) or PBS, 3 days per week during 6 weeks. Assessment of treatment effect was performed, based on clinical (weight, diarrhea, arthritis), histological (proximal and distal colon, caecum, ileum and tarsal/ankle joint) scores, and frequency of Treg in the spleen and lymph nodes (LN).

**Results:**

IL-2 administration had no effect on weight gain, either in B27- or NTG-rats. Over the 6 weeks of treatment, the clinical disease score worsened similarly in both IL-2-treated and control groups of B27-rats. The macroscopic and histological evaluation of gut and joints showed marked inflammation in B27-rats; however, no change related to IL-2 treatment was observed. In the B27-rats, the percentage of Treg was moderately increased after IL-2 treatment in the spleen, but neither in mesenteric nor peripheral LN in those rats.

**Conclusion:**

Our data demonstrate that a low dose of IL-2 administered before disease onset was moderately effective for boosting Treg but failed to prevent SpA development in B27-rat.

**Supplementary Information:**

The online version contains supplementary material available at 10.1186/s13075-021-02559-y.

## Background

Spondyloarthritis (SpA) is a chronic inflammatory disorder that predominantly affects the spine and sacroiliac joints. These typical features frequently combine with peripheral joint arthritis, enthesitis, and extra-articular manifestations, the most frequent of which being uveitis, psoriasis, and inflammatory bowel disease (IBD), including Crohn’s disease and ulcerative colitis [1]

Genetic predisposition underlying SpA is strong, the greatest contribution being attributable to the HLA-B27 allele. One of the most compelling evidences directly implicating HLA-B27 in the pathogenesis of SpA came from the animal model. Hence, rats transgenic for HLA-B27 and human β2-microglobulin (hβ2m) develop a spontaneous inflammatory disease that strikingly recapitulates the spectrum of SpA, by combining arthritis and spondylitis to ulcerative colitis and psoriasis [2]

In this model, we previously reported that altered dendritic cell (DC) function could play a critical role in triggering SpA since DCs from HLA-B27/hβ2m transgenic rats (B27-rats) promoted a biased expansion of pro-inflammatory Th17 cells, which might have a pathogenic role [3]. Furthermore, the altered interaction between DC and CD4+ T cells resulted in a modification of regulatory T cells (Tregs) function, consisting of decreased IL-10 and enhanced IL17 production by those cells [4].

Tregs that suppress effector T cell (Teff) proliferation and function play a critical role in maintaining peripheral tolerance and immune homeostasis. Disruption of the balance between Teff (including Th17) and Treg could be a key event in the pathogenesis of several autoimmune and inflammatory disorders including rheumatoid arthritis (RA), systemic lupus erythematosus, IBD, and SpA. Therefore, manipulating Treg in a way to restore immune balance is considered a new therapeutic concept for those disorders [5].

Interleukin (IL)-2 can promote the differentiation, survival, and function of Treg and help restoring the immune balance between Treg and Teff. Low-dose IL-2 has been reported to selectively expand Treg and inhibit Th17 cells and so has a broad therapeutic potential in immune-mediated inflammatory diseases, including rheumatoid arthritis, systemic lupus erythematosus, psoriasis, Crohn’s disease, and type 1 diabetes [6].

Given the dysfunction previously reported in Treg from B27-rats, we speculated that low-dose IL-2 administration could contribute to expand those cells, restore their anti-inflammatory potential, and prevent SpA development. Thus, in this study, we investigated the effect of low-dose IL-2 on the frequency of T cell subsets and its therapeutic efficacy on experimental SpA, in B27-rat.

### MethodsAnimals

The 33-3 line of SpA-prone B27-rats bearing 55 copies of HLA-B*2705 and 28 copies of hβ_2_m on a Fisher (F344) background were originally produced at the University of Texas Southwestern Medical Center (Dallas, TX). B27-rats and their nontransgenic (NTG) littermates were bred and maintained under conventional conditions. All animal procedures were approved by the institutional ethical committee (APAFIS-8910).

### IL-2 production and purification

Recombinant human (rh) IL-2 was produced in the CHO cell line by Sanofi® research division (Ferrara et al. 1987). The recombinant protein possesses a sugar moiety O-linked to the threonine residue at position 3 of the polypeptide chain, and sialic acid, galactose, and N-acetyl galactosamine are the components of this carbohydrate moiety, indicating that the recombinant molecule is identical to natural IL2. This IL-2 was produced in a fermentor with cells growing on microcarriers and perfusion of the defined medium. The supernatant was concentrated and purified. The rhIL2 protein batch, IL069P, was obtained after four chromatography purification steps and characterized. The protein showed a 99.8% purity with less than 0.3 EU/mg of endotoxin, a concentration of 881 μg/ml, a specific activity on murine cells of 18.3 10^6^ international unit (IU)/mg (as determined by cytotoxic T lymphocytes line CTLL2 assay) and complied with microbiological controls. The batch was stored at −20°C and the biological activity of this protein was confirmed in many different assays [7]. The rhIL-2 was previously shown as biologically active in the rat [8].

### Treatment with a low dose of rhIL-2

**Fig. 1:**
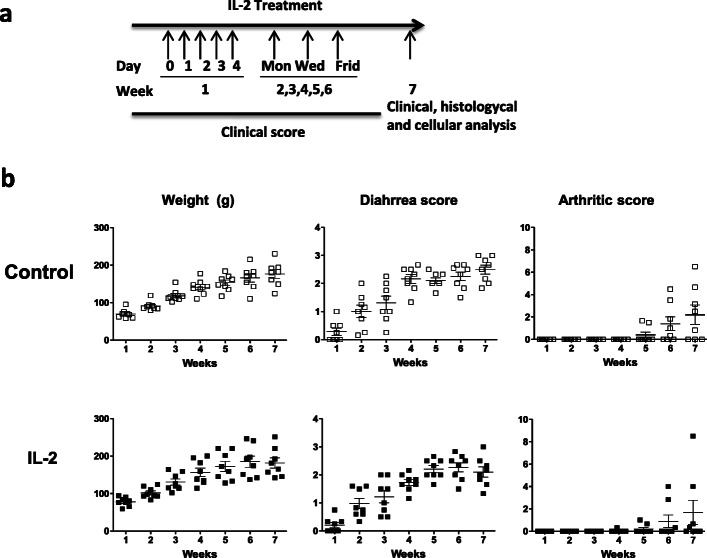
Effect of low-dose IL-2 treatment on SpA development in B27-rat. **a** Experimental schema of in vivo rhIL-2 treatment: B27-rats received i.p. 2000 IU of rhIL-2, on days 0, 1, 2, 3, and 4 during the first week, then 3 times a week for five consecutive weeks. Control B27-rats were treated with vehicle (PBS). **b** Weekly evolution of the clinical parameters in both groups of B27-rats over the treatment period, including weight, diahrrea, and arthritis. Horizontal bars indicate the mean from 8 rats/group

After determining which low dose of IL-2 was most appropriate in the preliminary experiment, age- and sex-matched B27-rats (8 animals/group) and NTG littermates (5–6 animals/group) were randomized 3 days before starting treatment with a permutation table (each cage contained at least one rat from each experimental group) to receive rhIL-2 (2,000 IU/injection) diluted in phosphate-buffered saline (PBS) or PBS only (0.4ml/injection). RhIL-2 was administered intraperitonally (i.p.) daily during the first week, then 3 days per week for 5 consecutive weeks (Fig. 1A). At week 7, the rats were sacrificed: the weight and length of the colon were measured and organs were harvested.

**Table 1:** Effect of low dose IL-2 treatment on intestinal histopathologic score in NTG and B27-rats

Histopathologic score (mean ± SEM)				
	Ulceration	Infiltration	Abcesses	Total score
**Proximal colon**				
**PBS-NTG**	0,2+0,2	0,8+0,2	0+0	**1,0**
**IL-2-NTG**	0,5+0,4	0,7+0,2	0,12+0,04	**1,3**
**PBS-B27**	2,6+0,17	2,6+0,1	0,3+0,12	**5,5**
**IL-2-B27**	2,5+0,2	2,6+0,1	0,3+0,14	**5,4**
**Distal colon**				
**PBS-NTG**	0,5+0,23	0,5+0,21	0+0	**1,0**
**IL-2-NTG**	1,2+0,76	1,3+0,36	0,1+0,1	**2,5**
**PBS-B27**	2,3+0,15	1,9+0,3	0,3+0,1	**4,6**
**IL-2-B27**	2,3+0,31	2,0+0,3	0,2+0,09	**4,4**
**Caecum**				
**PBS-NTG**	0,2+0,2	0,8+0,2	0+0	**1,0**
**IL-2-NTG**	0,5+0,4	0,7+0,2	0,12+0,04	**1,3**
**PBS-B27**	2,6+0,17	2,6+0,1	0,3+0,12	**5,5**
**IL-2-B27**	2,5+0,2	2,6+0,1	0,3+0,14	**5,4**

### Clinical score

The rats were examined three times a week in a blind fashion for clinical symptoms of colitis (loose stools and/or frank diarrhea, proctitis), arthritis (erythema and/or swelling), and alopecia and were assigned for each of these symptoms a score on a scale ranging from 0 (normal) to 4, as previously described [9]. The average daily total of these scores was calculated each week (maximum score per rat: 15). Body weight was monitored weekly.

### Histology

Upon sacrifice, tissue samples were fixed in 4% phosphate-buffered formalin embedded in paraffin. Sections (4μm) were prepared and stained with hematoxylin-eosin. Microscopic lesions of the colonic mucosa were semi-quantitatively scored following criteria previously described [9] and shown in Supplementary figure 1. The paws of rats were excised and fixed in 4% phosphate-buffered formalin. After 24 h of fixation, they were immersed for 3 weeks in Decal solution (Serva). After decalcification, they were sectioned and stained with hematoxylin and eosin. Arthritis severity was quantified, based on synovial hyperplasia, pannus formation, inflammatory cell infiltrate of niches, and destruction of articular cartilage and bone (Supplementary figure 1). Scores were assigned twice blindly by two different investigators. Results represent the mean of all the scores.

### Regulatory and effector T cell analysis

The frequency of Treg and Teff was determined in splenocytes and LN using FITC-conjugated anti-CD25 (clone OX-39), and PE-anti-FoxP3 (clone FJK-16s), antibodies from BD Pharmingen. FoxP3 staining was done according to the manufacturer’s instructions (Thermofisher). Labeled cells were analyzed with FACS Fortessa (BD Biosciences). For intracellular cytokine staining, LN and spleen cells were incubated for 4 h with phorbol myristate acetate (PMA; 10 ng/ml), ionomycin (1 μg/ml), and brefeldin A (2 μg/ml). All data were analyzed using FlowJo software (Tree Star).

### Statistical analysis

Data are expressed as the mean ± SEM. Multiple comparisons between groups were determined using a 2-way analysis of variance (ANOVA) with Tukey’s test. *P* values < 0.05 were considered significant.

## Results

### Determination of the low dose of IL-2 to be administered as a treatment to B27-rats

To determine the most appropriate rhIL-2 dose to be used in the subsequent therapeutic trial, we performed two consecutive preliminary experiments to determine the in vivo impact of IL-2 on T cell subsets. First, we administered i.p. a wide range of IL-2 doses (100,000; 50,000; 25,000 or 5,000 IU/rat) or vehicle only, daily for five consecutive days, to 4-week-old B27-rats. Mesenteric LN and spleen were harvested on day 7 to analyze the ratio of Teff/Treg, IL-17/IL-10 cytokine production, and expression of Treg markers. The highest doses of IL-2 (100,000, 50,000, and 25,000 IU) induced expansion of both Treg and Teff (data not shown). To maximize the selectivity of the IL-2 effect for Treg, using a similar treatment schema as in the first experiment, we next performed a second experiment using low doses of IL-2 (5000 or 2000 I/rat), as shown in Supplementary figure 2 and 3. In this experiment, both doses of rhIL-2 induced an important expansion of cells in mesenteric LN without alteration of the proportion of Treg and Teff, but no change in the spleen cellularity. Most interestingly, the ratio of pro-inflammatory (IL-17) over anti-inflammatory (IL-10) cytokines was reduced in the LN with the smallest dose of rhIL-2, whereas it was the case in the spleen with the highest dose (Supplementary figure 2 and 3). There was a trend towards an increased expression of cell surface Treg markers (CTLA-4, LAG-3, and GITR) in the spleen, specially with the 2000 IU dose that was not observed in LN (Supplementary figure 2 and 3). Thus, we selected the lowest dose of rhIL-2 (2000 U) to be used in further study, considering that the most important objective was to restore the cytokine balance in Treg in favor of IL-10 in the lymphoid organs draining inflammatory sites.

### Treatment with a low dose of IL-2 failed to prevent colitis development in B27-rat

**Fig. 2:**
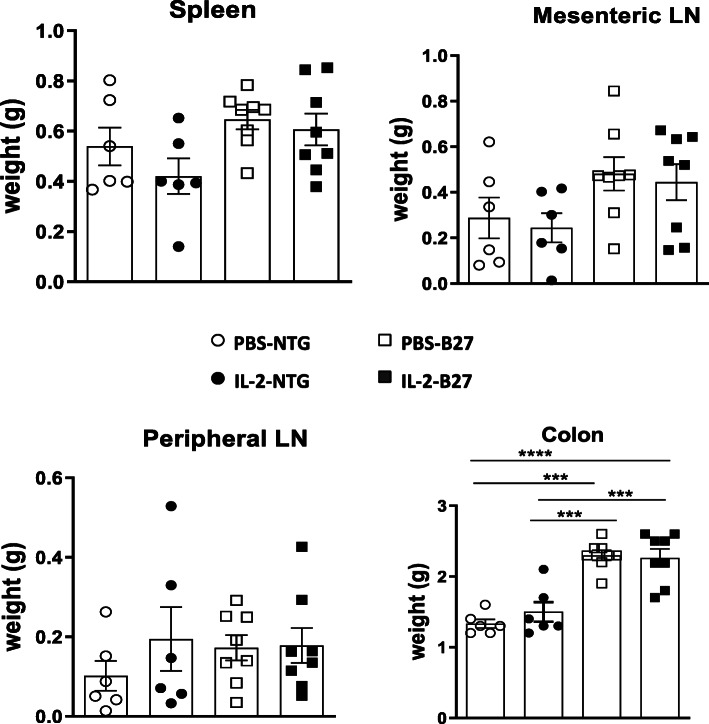
Effect of low-dose IL-2 treatment on lymphoid organs and colon weight. Groups of NTG and B27-rats were treated with vehicle (PBS) or rhIL-2 for 6 weeks. At week 7, the weight of lymphoid organs and colon was determined after necropsy. Values are the mean ± SEM of 5–8 animals/group. **p < 0.005, ***p < 0.0005

**Fig. 3:**
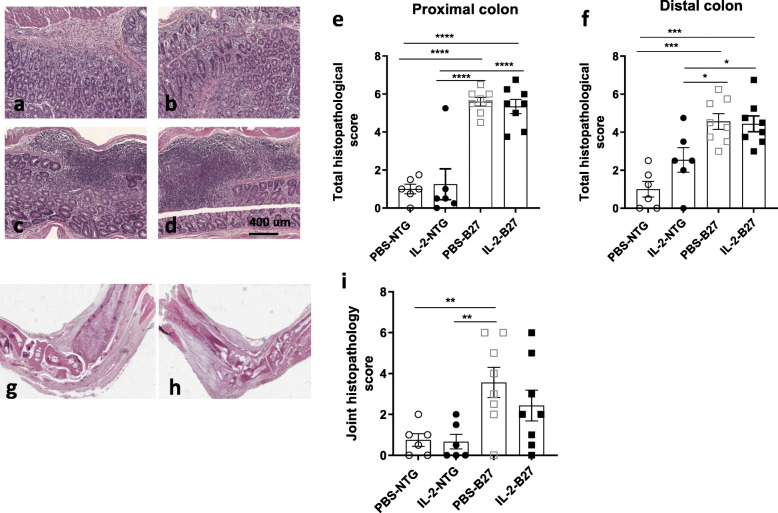
Colon and paw histopathology in B27- and NTG rats treated with low-dose rhIL-2. Proximal colon of B27-rats treated with **a** PBS or **b** low-dose IL-2. Distal colon of B27-rats treated with **c** PBS or **d** low-dose IL-2. Original magnification x10. Graphs of total histopathological score of **e** proximal and **f** distal colon in different groups of rats. Histopathology of hindpaws from B27-rats treated with **g** PBS or **h** IL-2 and stained with hematoxylin and eosin. **i** The total histopathological score is shown in the graph. Values are the mean ± SEM of 5–8 animals/group. **p < 0.005. Values are the mean ± SEM . n= 5–8 animals/group. *p < 0.05, ***p<0.0005, ****p<0.0001

To investigate the in vivo modulatory effect of a low dose of IL-2 treatment on SpA development, premorbid B27-rats and control NTG rats were treated with rhIL-2 or vehicle (PBS), for 6 consecutive weeks (Fig. 1a). As expected, PBS-treated B27-rats developed colitis symptoms, characterized by chronic diarrhea that progressively worsened over the treatment period. B27-rats receiving low-dose IL-2 developed diarrhea of comparable severity, without a significant difference as compared to the PBS-treated group (Fig. 1b). In addition, increased colonic weight (Fig. 2) and histological inflammation (Fig. 3a–d and Table 1) corroborated clinical manifestations. Both low-dose IL-2- and PBS-treated B27-rats showed similar severity of gut inflammation that was characterized by ulcerations, loss of goblet cells, and tissue disruption by histology (Fig. 3a–d and Table 1). The colonic inflammation score was significant increased in B27-rats treated or not with low-dose IL-2, as compared to NTG rats (Fig. 3e, f). The NTG rats, whether treated or not with IL-2, remained healthy, clinically, and by histology (data not shown)**,** thereby indicating that IL-2 administration had no toxic effect. Altogether, those results indicate that low-dose IL-2- exerted no influence on colitis development in B27-rat.

### Low-dose IL-2 treatment did not significantly alter arthritis development in B27-rat

Concerning arthritis development, both PBS-or IL-2-treated B27-rats developed clinical joint inflammation during the observation period that was not significantly different between both groups (Fig. 1b). Neither was the histopathological score significantly different between those groups (Fig. 3g, i). However, there were fewer arthritic rats in the IL-2-treated group at the end of the study period (2/8 in IL-2 vs. 4/8 in the PBS-treated group; Fig. 1b) and joint histopathology appeared somewhat milder in the IL-2-treated group (Fig. 3i). NTG rats had a normal joint both clinically (data not shown) and by histology (Fig. 3i).

### Evolution of Treg frequency after IL-2 treatment in B27-rat

**Fig. 4:**
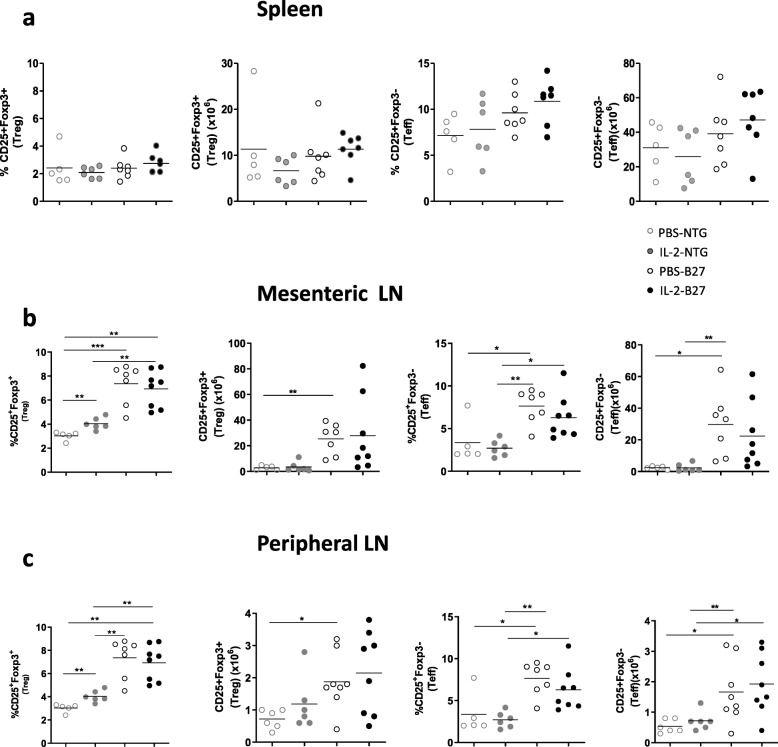
Low-dose IL-2 fails to expand Tregs in B27-rat. The graphs represent the percentage and absolute numbers of Treg and Teff in spleen (**a**), mesenteric (**b**), and peripheral (**c**) LN, collected at time of sacrifice. Values are the mean of 5–8 rats/group. *p<0.05, **p< 0.005, ***p<0.0005

**Fig. 5:**
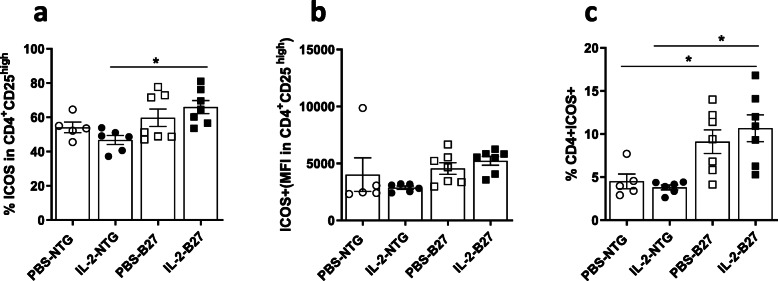
Low-dose IL-2 did not modify ICOS expression in Treg. The graphs represent the percentage (**a**) and MFI (**b**) of ICOS expressed on Treg and the percentage of CD4+ICOS+ T cells among mesenteric LN cells (**c**), collected at time of sacrifice. Values are the mean of 5–8 rats/group. *p<0.05

We investigated the consequence of IL-2 treatment on the proportion and absolute number of Treg and Teff in the spleen, mesenteric, and popliteal LN isolated from PBS- and IL-2-treated rats at the time of sacrifice (Fig. 4a–c). There was no significant difference in the proportions of Treg and Teff in the spleen between B27- and NTG rats, whether treated or not with IL-2 (Fig. 4a). In contrast, both populations of Treg and Teff were increased in mesenteric and popliteal LN, in B27-rats, as compared to NTG rats (Fig. 4b, c). Regarding rhIL-2 effect, it resulted in a significant increase of Treg both in mesenteric and peripheral LN only in NTG-treated rats. Moreover, we observed a slight but non-significant decrease of Teff frequency in IL-2-treated B27-rats. We previously demonstrated a dysregulated production of IL-10 and IL-17 by Treg involving ICOS signaling. In this study, IL-2 treatment did not significantly alter ICOS expression in mesenteric LN CD4^+^ T cells, including Treg (Fig. 5a–c).

## Discussion

Treg plays a central role in the induction and maintenance of immune homeostasis in vivo as they suppress almost every kind of Teff, including Th17 cells. We previously demonstrated in B27-rats altered crosstalk between DCs and T cells, that induces a modification of Treg function, resulting in decreased IL-10 and enhanced IL17 production by those cells [4]. Thus, we surmised that such alteration could be a key element in the emergence of Th17-driven pathology observed in these rats.

Using this experimental SpA model, we previously tried to overcome the deficiency of the IL-10 production or to control the excess of the IL17 production, by administering either recombinant IL-10 or IL-17 neutralizing antibody, respectively [9] [3],. However, those treatments failed to counter the development of rat-SpA. Such negative results prompted us in the present study to try enhancing the differentiation, development, and/or functional activity of Treg, rather than directly inhibiting the function of Teff.

Several studies have suggested that Treg plays a role in the etiology of SpA [10–12]. Surprisingly, it was described that SpA patients had an elevated frequency of circulating Foxp3+ Tregs that could be reversed upon anti-TNF biotherapy treatment [13, 14]. This suggested that SpA patients could upregulate Treg in response to inflammation. This observation raised the question of why Treg was unable to suppress aberrant inflammation. A possible explanation is that Treg in patients with SpA is functionally altered, similar to our observations in B27-rat. Consistent with this hypothesis, in patients with ankylosing spondylitis, peripheral blood Treg was characterized by low Foxp3 expression, little STAT5 phosphorylation upon IL-2 stimulation, and more extensive methylation in the CNS2 region of the *FOXP3* gene, confirming that functional defects of Treg are present in SpA [15].

Low-dose IL-2 has been reported to selectively expand and activate Treg and so has a broad therapeutic potential by regulating the immune balance of T cells in several disorders [16]. Here, we tested for the first time the possibility to impact experimental SpA by administration of low-dose IL-2 in B27-rats which the aim of inducing Treg-expansion/activation.

However, low-dose IL-2 administered to B27-rats was inefficient to alter SpA development. The low dose of IL-2 (2000 iU) used in our work induced slight changes in Treg and Teff in those rats. Overall, we observed no change in Teff nor in Treg in the spleen and a mild decrease of Teff in LN, without a significant effect on Treg frequency.

The identification of a dose of IL-2 capable of safely reversing the Treg/Teff balance in favor of Treg is of major importance. The trials of low-dose IL-2 reported so far have used different doses and different schedules of administration. However, their main conclusions are that IL-2 induces important expansion of Treg and that their maximum range and duration are dose-dependent [17]. Consistent with these findings*,* Rosenzwajg et al. analyzed the kinetics and dose-relationship of IL-2 treatment on Treg frequency in type 1 diabetes patients. They observed that low-dose IL-2 therapy induced a dose-dependent increase in CD4+Foxp3+ and CD8+Foxp3+ Treg numbers and proportions, the duration of which was markedly dose-dependent [18].

Haizhuan et al. demonstrated that IL-2 could increase the frequency of peripheral blood CD4+ Treg, CD8+ Treg, and Th17 cells and that Treg increase was stronger than that of Th17 cells, consequently correcting the imbalance observed in SpA patients. It is important to emphasize that in this paper, the authors analyzed the short time (5 consecutive injections) effect of low-dose IL-2 on peripheral Treg frequency [19].

Previously, we reported that an accumulation of Teff cells over Treg in mesenteric LNs from B27-rats occurred in parallel with disease development [4]. Low-dose IlL2 (2000 IU) used in our study was able to increase significantly the frequency of Treg only in NTG rats. Thus, the selected regimen was insufficient to reverse Teff/Treg imbalance in B27-rats. In our preliminary experiments, higher doses of IL-2-ranging from 25,000 to 100,00 UI/rat induced an expansion of Teff as well as Treg and were unlikely to be more efficacious. However, it is still possible that an intermediate dose of IL-2 (5000–10,000 IU/rat) would have been more efficacious. Consistent with this interpretation, Goettel et al. demonstrated that administration of 10,000 IU of IL-2/day ameliorated colitis in a humanized mouse model associated with the expansion of Treg in the blood and spleen but not in mesenteric LN nor in the colon [20].

Similar to SpA and other inflammatory disorders, the immune imbalance between Teff and Treg is critically involved in the pathogenesis of RA. Rosenzweig et al. performed a single open-label clinical trial with low-dose IL-2 therapy in 46 patients with autoimmune disease (including 4 patients with RA). All patients received low-dose IL-2 (10^6^ IU/day) for 5 days and followed by an injection every 2 weeks for 6 months. With this dose and treatment scheme, Tregs were selectively activated and expanded without impact on the level of Teff [16]. More recently, it was proven for the first time that low-dose IL-2 (10^5^ IU/3 days) reduced the severity of vascular and bone lesions in collagen-induced arthritis by inhibition of osteoclast formation through Jun kinase-dependent pathway [21].

## Conclusions

Negative results of this preclinical low-dose IL-2 trial in the B27-rat model, considered as one of the most relevant animal models of SpA, underscore the complexity of SpA mechanisms and suggest that combining different treatments could be a more effective therapeutic strategy than targeting a single pathway in this disorder.

## Supplementary information

**Additional file 1:.** Supplementary figure 1. Scores used for histopathological assessment of intestinal and articular lesions. Supplementary figure 2. Impact of administration of low-dose rhIL-2 on Treg. B27-rats (n = 3/group) were injected i.p. daily for 5 consecutive days with PBS, 2,000 or 5,000 IU rhIL-2. At day 7, (a) the total number of cells, (b) the frequency of Treg, (c) the frequency of Teff, (d) ratio of Teff to Treg, (e) IL-10, (f) IL-17 intracellular production, (g) ratio of IL-17 to IL-10 intracellular cytokines production by Tregs, (h) Treg markers expression in LN from B27-rats were determined. Values represent the mean of 3 rats/group. *p < 0.05. Supplementary figure 3. Impact of administration of low-dose rhIL-2 on Treg. B27-rats (n = 3/group) were injected i.p. daily for 5 consecutive days with PBS, 2,000 or 5,000 IU rhIL-2. At day 7, (a) the total number of cells, (b) ratio of Teff to Treg, (c) ratio of IL-17 to IL-10 intracellular cytokines production by Tregs and (d) Treg markers expression in spleen from B27-rats were determined. Values represent the mean of 3 rats/group. *p < 0.05

## Data Availability

All data relevant to the study are included in the article.
