# Supplementary material for: Effects of a low-dose IL-2 treatment in HLA-B27 transgenic rat model of spondyloarthritis
Source: Arthritis Res Ther. 2021 Jul 16;23:193. doi: 10.1186/s13075-021-02559-y (PMC8283890; doi:10.1186/s13075-021-02559-y)
Supplement: Supplementary file 1 — Additional file 1:. Supplementary figure 1. Scores used for histopathological assessment of intestinal and articular lesions. Supplementary figure 2. Impact of administration of low-dose rhIL-2 on Treg. B27-rats (n = 3/group) were injected i.p. daily for 5 consecutive days with PBS, 2,000 or 5,000 IU rhIL-2. At day 7, (a) the total number of cells, (b) the frequency of Treg, (c) the frequency of Teff, (d) ratio of Teff to Treg, (e) IL-10, (f) IL-17 intracellular production, (g) ratio of IL-17 to IL-10 intracellular cytokines production by Tregs, (h) Treg markers expression in LN from B27-rats were determined. Values represent the mean of 3 rats/group. *p < 0.05. Supplementary figure 3. Impact of administration of low-dose rhIL-2 on Treg. B27-rats (n = 3/group) were injected i.p. daily for 5 consecutive days with PBS, 2,000 or 5,000 IU rhIL-2. At day 7, (a) the total number of cells, (b) ratio of Teff to Treg, (c) ratio of IL-17 to IL-10 intracellular cytokines production by Tregs and (d) Treg markers expression in spleen from B27-rats were determined. Values represent the mean of 3 rats/group. *p < 0.05 [file 13075_2021_2559_MOESM1_ESM.pdf]

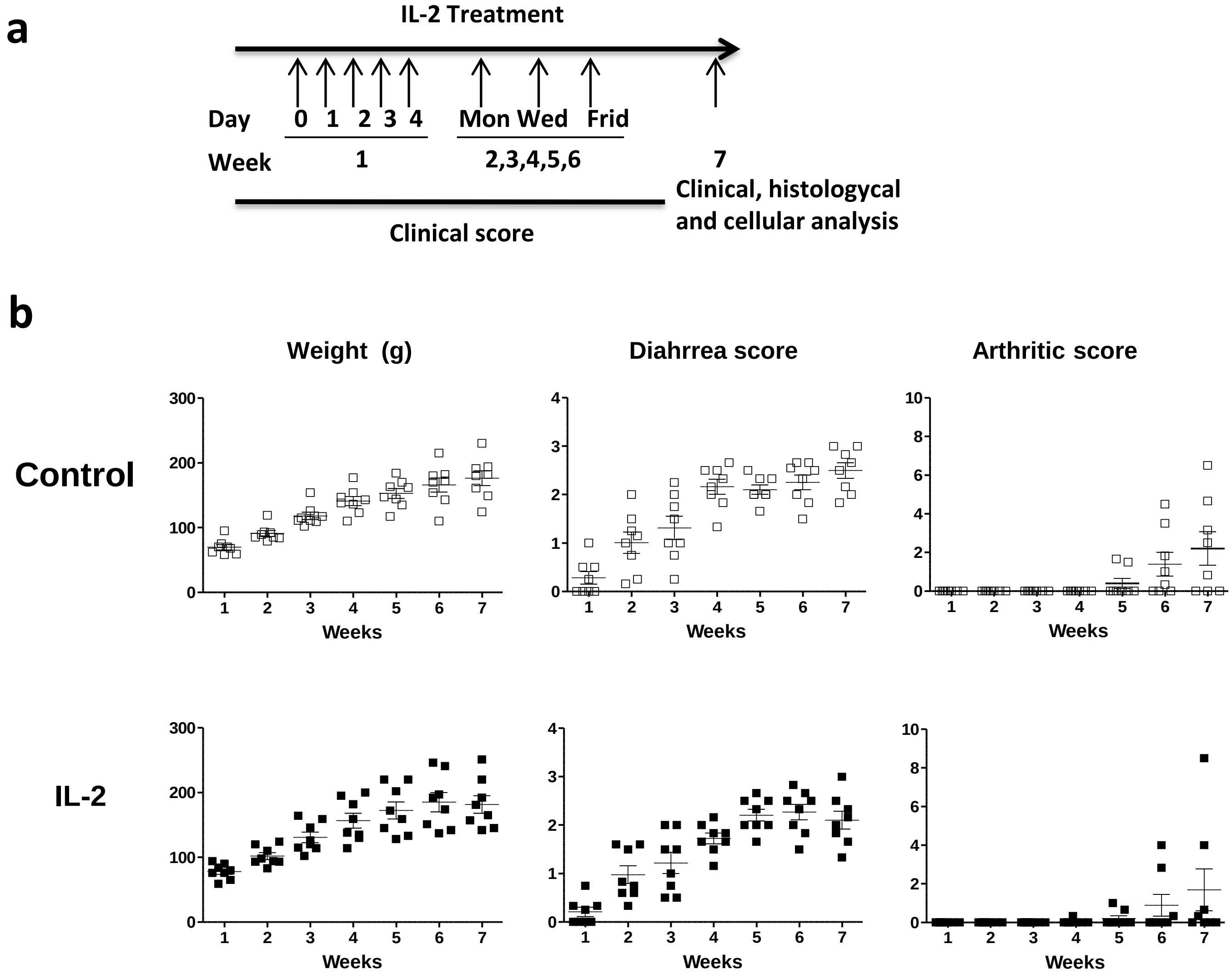

Araujo, et al, Figure 1

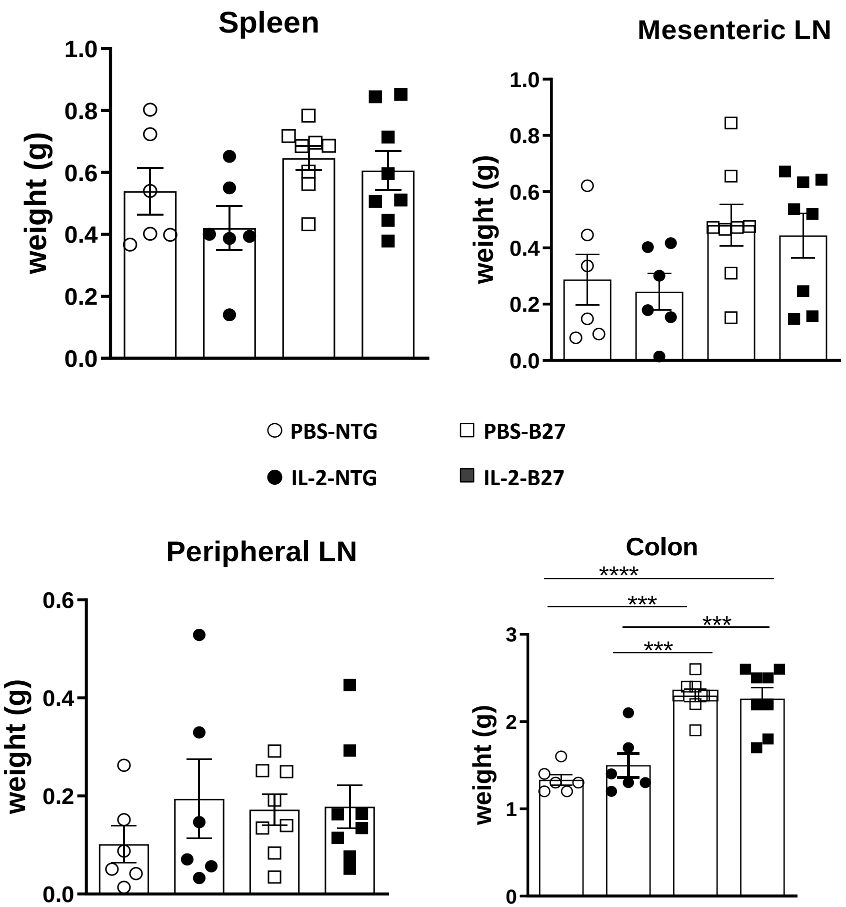

Araujo, et al, Figure 2

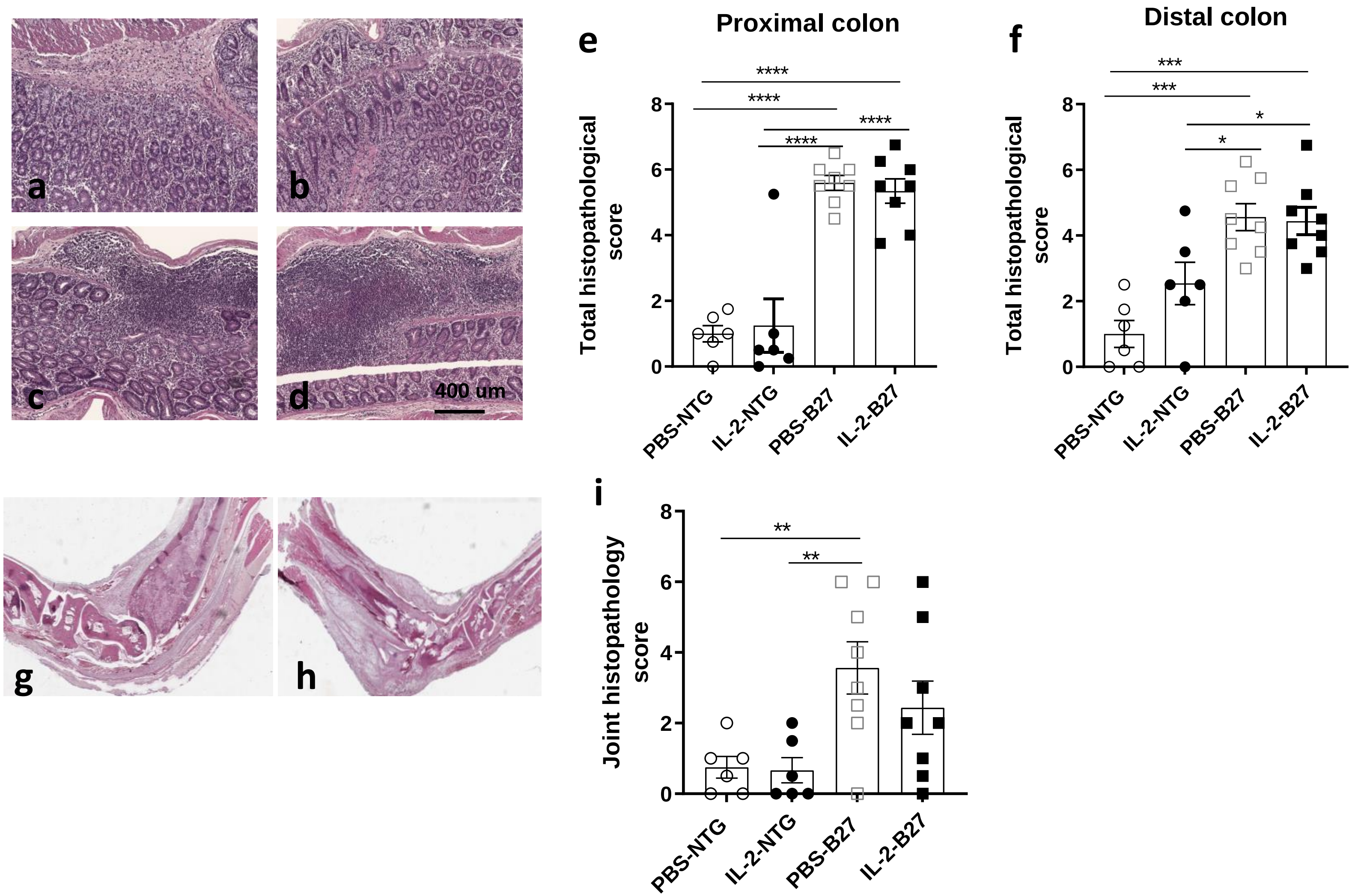

Araujo, et al, Figure 3

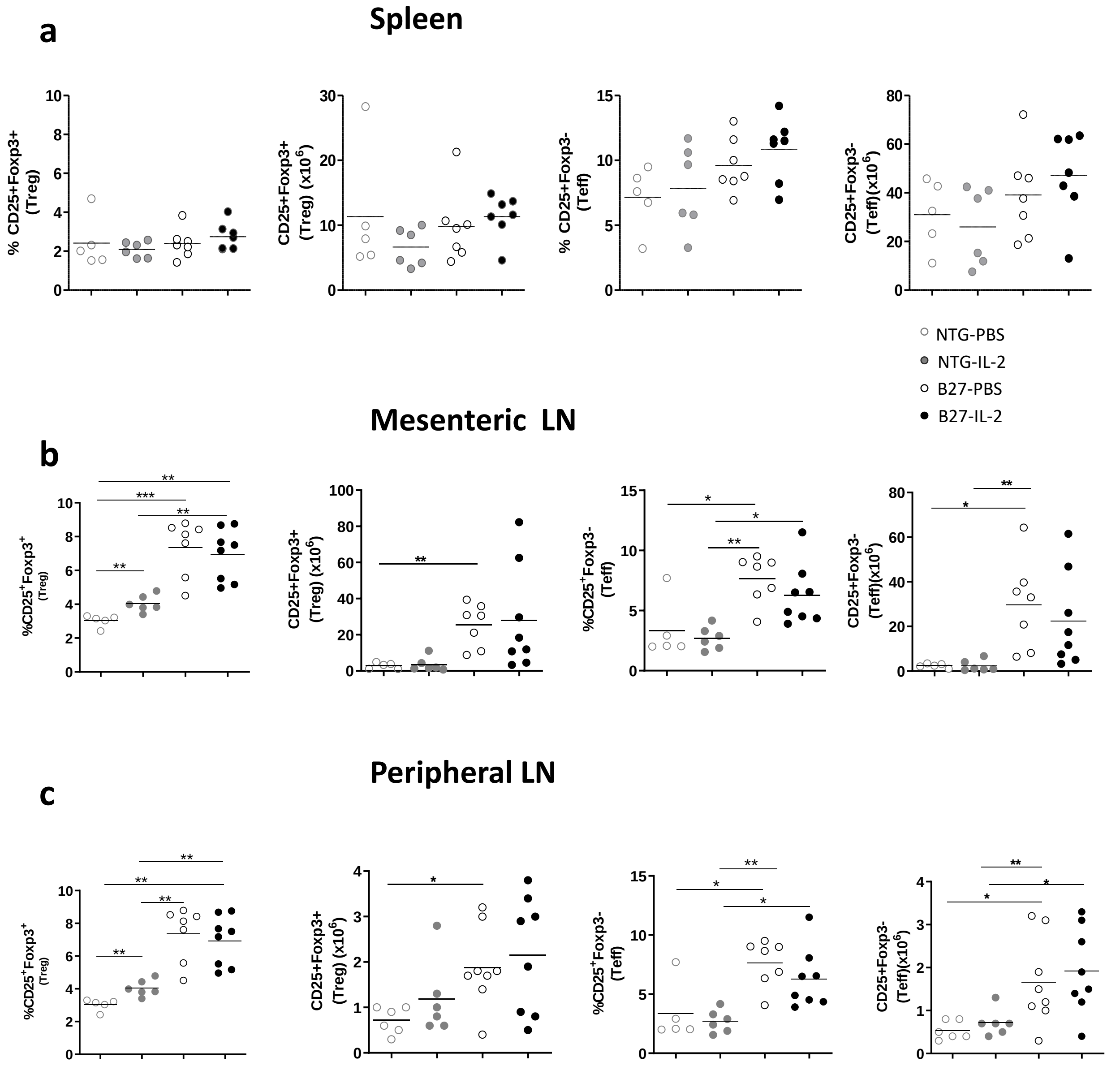

Araujo, et al, Figure 4

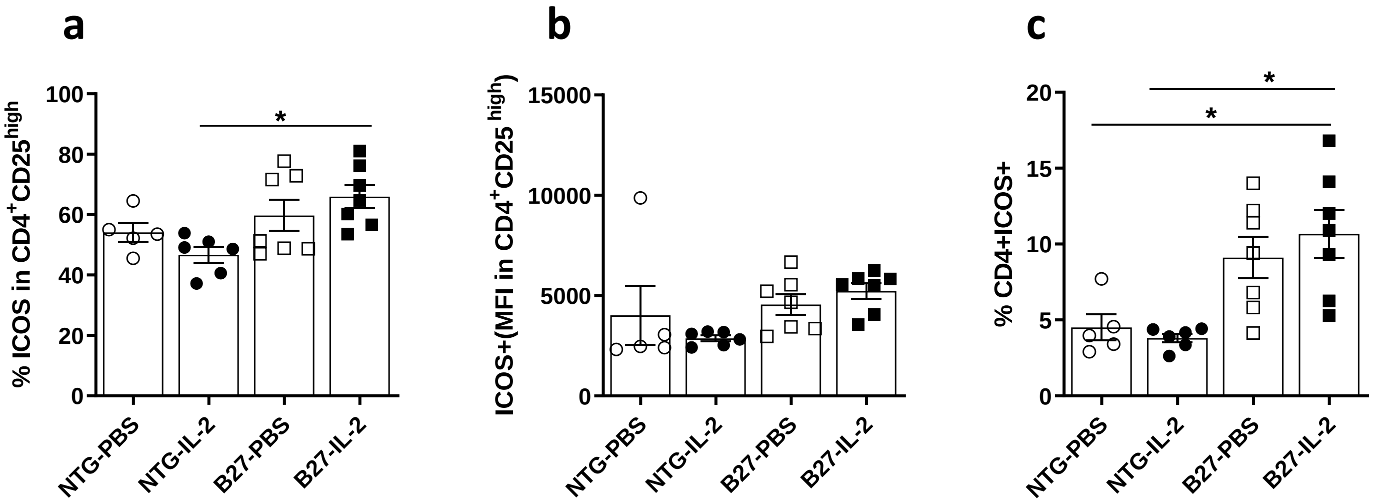

Araujo, et al, Figure 5

Scores used for histopathological assessment of intestinal and articular lesions

Intestin		Score
**Ulceration**	absent	0
	< 1/3	1
	> 1/3	2
	> 2/3	3
**Cellular infiltration**	Absent	0
	Mucosa	1
	Sub-mucosa	2
	Muscularis	3
**Crypt abscesses**	Absent	0
	Present	1

Articulation		Score
**Synovial tickness**	None	0
	Mild	1
	Moderate	2
	Severe	3
**Infiltration of niches**	None	0
	Mild	1
	Moderate	2
	Severe	3

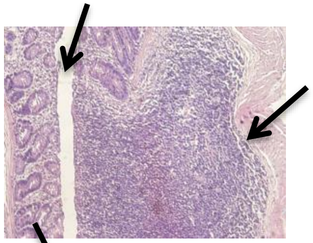

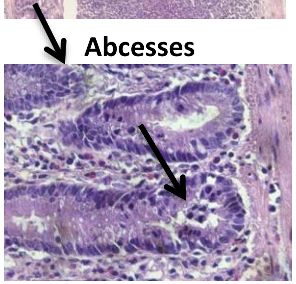

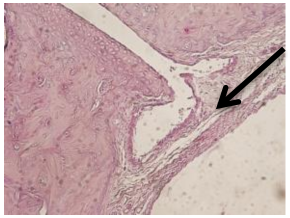

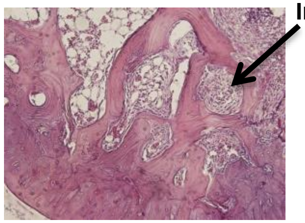

Araujo, et al, Supplementary figure 1

Mesenteric LN

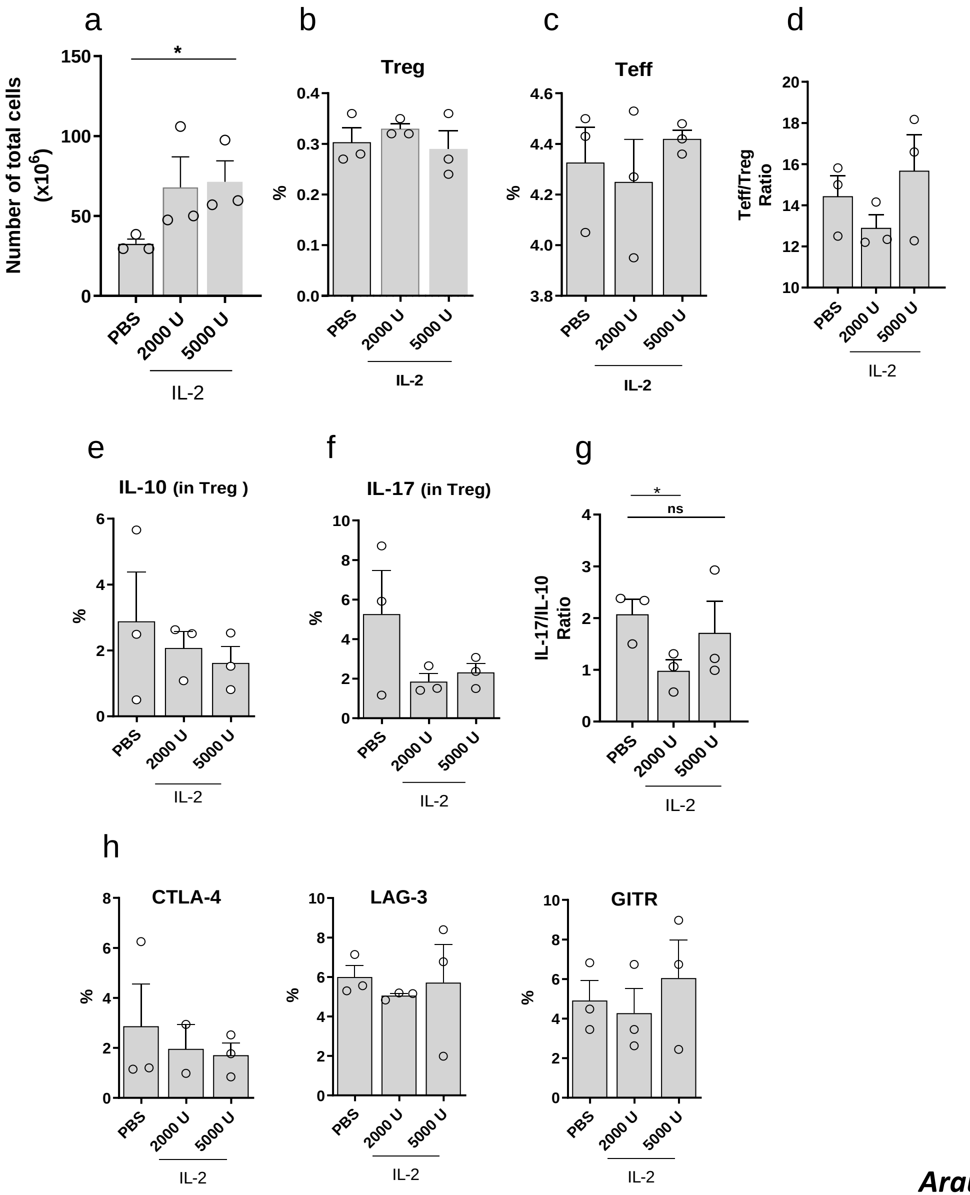

Araujo, et al, Supplementary figure 2

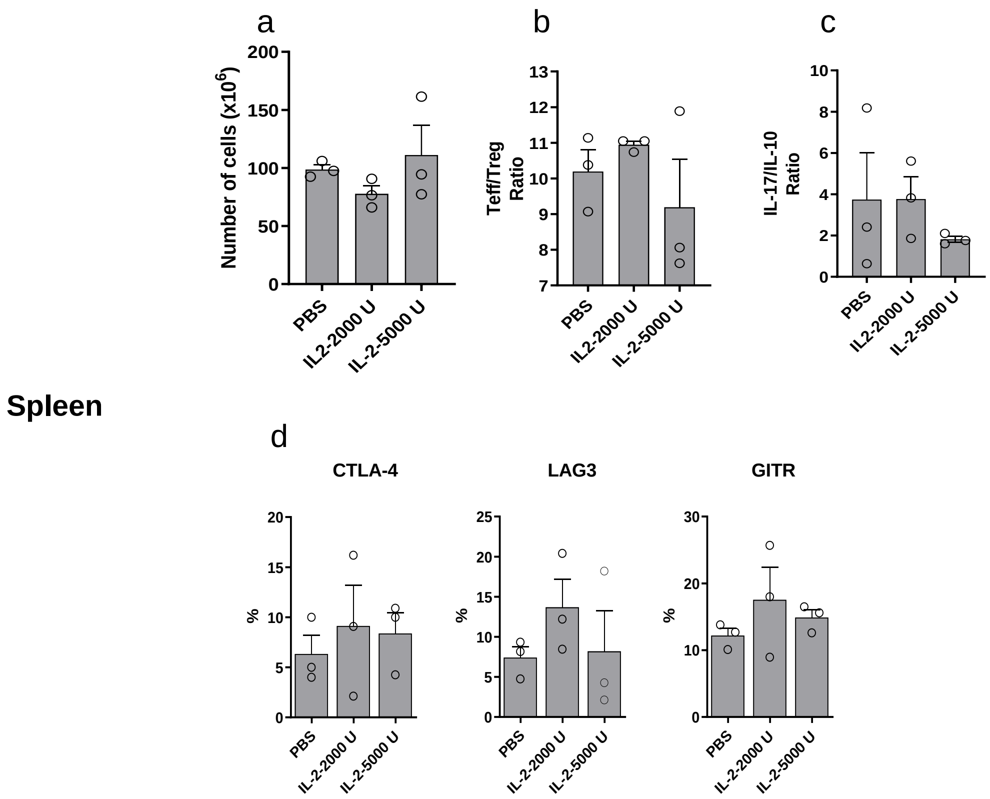

Araujo, et al, Supplementary figure 3

Table 1. Effect of low dose IL-2 treatment on intestinal histopathologic score in NTG and B27-rats.

	Histopathologic score (mean ± SEM)			
	**Proximal Colon**			
	**Ulceration**	**Infiltration**	**Abcesses**	**Total score**
**NTG-PBS**	0,2±0,2	0,8±0,2	0±0	**1,0**
**NTG-IL-2**	0,5±0,4	0,7±0,2	0,12±0,04	**1,3**
**B27-PBS**	2,6±0,17	2,6±0,1	0,3±0,12	**5,5**
**B27-IL-2**	2,5±0,2	2,6±0,1	0,3±0,14	**5,4**
	**Distal Colon**			
	**Ulceration**	**Infiltration**	**Abcesses**	**Total score**
**NTG-PBS**	0,5±0,23	0,5±0,21	0±0	**1,0**
**NTG-IL-2**	1,2±0,76	1,3±0,36	0,1±0,1	**2,5**
**B27-PBS**	2,3±0,15	1,9±0,3	0,3±0,1	**4,6**
**B27-IL-2**	2,3±0,31	2,0±0,3	0,2±0,09	**4,4**
	**Caecum**			
	**Ulceration**	**Infiltration**	**Abcesses**	**Total score**
**NTG-PBS**	0,2±0,2	0,8±0,2	0±0	**1,0**
**NTG-IL-2**	0,5±0,4	0,7±0,2	0,12±0,04	**1,3**
**B27-PBS**	2,6±0,17	2,6±0,1	0,3±0,12	**5,5**
**B27-IL-2**	2,5±0,2	2,6±0,1	0,3±0,14	**5,4**
